# Validation of daily increment formation in otoliths for *Gymnocypris selincuoensis* in the Tibetan Plateau, China

**DOI:** 10.1002/ece3.1572

**Published:** 2015-07-15

**Authors:** Chengzhi Ding, Yifeng Chen, Dekui He, Juan Tao

**Affiliations:** 1 Laboratory of Biological Invasion and Adaptive Evolution, Institute of Hydrobiology, Chinese Academy of SciencesWuhan, 410072, China; 2 Asian International Rivers Center, Yunnan UniversityKunming, 650091, China; 3 University of Chinese Academy of SciencesBeijing, 100049, China

**Keywords:** Daily increment, *Gymnocypris selincuoensis*, Otolith, Tibetan Plateau, validation

## Abstract

Daily increment validation in fish otolith is fundamental to studies on fish otolith microstructure, age determination and life history traits, and thus is critical for species conservation and fishery management. However, it has never been done for Schizothoracine fish, which is the dominant component of fish fauna in the Tibetan Plateau. This study validated the daily increment formation of *Gymnocypris selincuoensis*, as a representative of Schizothoracine fish, by monitoring the growth of hatchery-reared larvae group and wild-caught post-yolk-sac larvae group under controlled experiments. The results from monitoring the hatchery-reared larvae group showed that sagittae and lapilli were found in yolk-sac larvae, and formed 5–7 days before hatching, but asterisci were not found until 11 days post-hatching. The first increment in sagittae and lapilli was formed in the first day after hatching. The daily periodicity of increment formation was examined and confirmed in sagittae and lapilli of both larvae groups. However, sagittae were better for age determination than lapilli for larvae at earlier days. For larval *G. selincuoensis* older than 50 days, lapilli were the only otolith pair suitable for larvae daily age determination. This study validated the daily increment formation in Schizothoracine fish for the first time has primary implications to other fishes from this subfamily.

## Introduction

Daily increment validation in fish otolith is fundamental to studies on fish otolith microstructure, age determination and life history traits, and thus is critical for species conservation and fishery management (Campana and Neilson 1985; Jia and Chen 2009). The earliest age estimation using otoliths began with the observation of annul rings on otoliths (Jackson 2007). Since Pannella (1971) first found and described the evidence of daily growth increments in otoliths, it is now regarded as a widespread phenomenon in teleost fishes. Daily increment has been applied in age determination for larvae in days and juveniles, and is critical to study the early life history of fish (Campana and Neilson 1985).

Daily increments in otoliths are caused by endogenous circadian endocrine rhythms which arose from periodic environmental factors, such as daily photoperiod and water temperature fluctuation (Radtke and Dean 1982; Campana and Neilson 1985). Although the utility of daily growth increments in providing estimates of age have been validated in a wide variety of species, the increment deposition in otoliths is varied among different species (Campana and Neilson 1985; Morales-Nin 2000; Nakaya et al. 2008; Carvalho et al. 2014). In addition, within the same species, microstructural characteristics and the frequency of deposition can be influenced by development stages, environmental variables, nutritional intake, and growth rate (Neilson and Geen 1982; Campana 1983; Fukuda et al. 2009). Therefore, validation of the time of the first increment formation and the periodicity of increments are essential for ageing fish and studying their life history traits (Campana and Neilson 1985).

Schizothoracine fishes belong to the family of Cyprinidae. They are dominant fishes in rivers and lakes of the Qinghai-Tibetan Plateau and surrounding regions (Cao et al. 1981). In addition, due to the increasing influence of global climate change and anthropogenic activities on aquatic ecosystems in this area, scientists have attached great attention to Schizothoracine fish (He and Chen 2006; Jia and Chen 2009; Li et al. 2009; Tao et al. 2015). However, daily increment validation has never been done for Schizothoracine fish, thus hinder the related species conservation and fishery management. *Gymnocypris selincuoensis* (Cyprinidae: Schizothoracinae) is the only species of Schizothoracine fishes living in Lake Selincuo, a high-altitude lake (4530 m) which located in the north of Qinghai-Tibet Plateau. It is characterized as a slow-growing, late-maturing, long-lived (>25 years) cyprinid which can reach up to 43 cm in length, 1.1 kg in weight (Chen et al. 2002a, 2004). However, as a representative fish species of Schizothoracinae fishes that has been well studied, neither validation of the time of the first increment formation nor the daily periodicity of micro-increment formation in otoliths have been validated to date. Existing studies on this species only have proved the formation time of annuli and its periodicity, and described characteristics of annuli (Chen et al. 2002b,c). Therefore, the objectives of the present study were to validate: (1) the time of the first increment formation, (2) and the periodicity of increment formationin otoliths of *G. selincuoensis* larvae.

## Materials and Methods

### Experimental design

Two experiments were conducted in captivity on the edge of Lake Selincuo in a field tent. Experiment 1 was with hatchery-reared larvae that were maintained in captivity for 10 days within the tent. Experiment 2 was with wild post-yolk-sac larvae that were marked with environmental stress due to the capture and transport process.

#### Experiment 1

**Figure 1:**
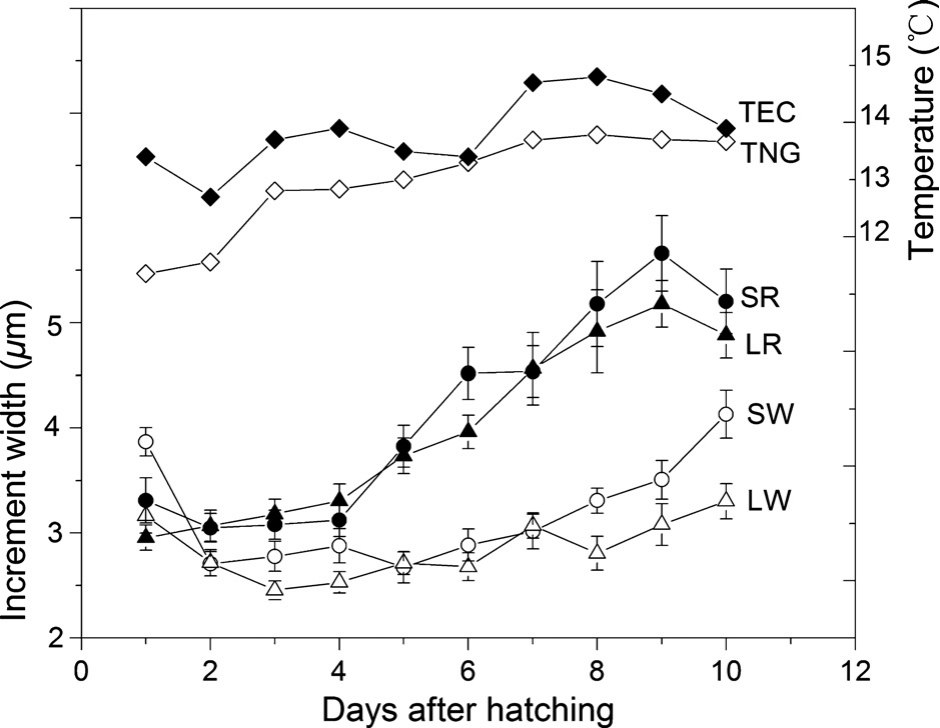
Temperatures of the experimental conditions (TEC) and the nursery ground (TNG) and mean ± SE width of otolith microincrements (Sagittae of wild larvae, SW; Sagittae of reared larvae, SR; Lapilli of wild larvae, LW; Lapilli of reared larvae, LR).

Two sexually matured female and two male fish were collected on May 29, 2010 to provide fertilized eggs by artificial insemination. Eggs hatched from June 11 to June 14, but only the larvae hatched on June 12 were used in the experiment. The tent ensured that early stages experienced ambient photoperiod and temperature, and water condition was maintained by a daily change of two-thirds the volume from nearby river water. Eggs were sampled daily to assess whether increment formation commenced before hatching. On each day of the first 10 days after hatching, 10 larvae were sampled. Daily water temperature was measured in the tank and in the wild nursery ground (TNG) of the Lake Selincuo. The temperature in the experimental tank was higher than the wild by 1–2°C (Fig. 1).

#### Experiment 2

The wild larvae were collected using hand net from Zageng Tsangpo River (31°48.77′N; 88°25.42′E), the primary tributary of Lake Selincuo on June 16th, 2010. A total of 106 larvae were sampled. The larvae were transported from the river within a 1 L bottle of river water to the field tent (31°46.27′N; 89°20.94′E). The transportation lasted 2 h during which water temperature rose from 9.7 to 14.3°C and dissolved oxygen concentration decreased from 5.9 to 5.1 mg L^−1^. It was assumed that the wild larvae otoliths were marked by the environmental stress associated with capture and transportation. After transporting them into the tent, the larvae were treated in the same experimental conditions as in Experiment 1. Larvae were fed twice daily with zooplankton collected from the river. On each of the first 5 days after marking, 10 larvae were sampled, and on sixth day, the remaining 56 larvae were sampled.

### Otolith preparation

Prior to otolith extraction, larvae from experiment 1 and 2 were measured to the closest 0.01 mm in total length (*T*_L_) and preserved with 70% ethanol (ETOH). The lapillus and sagitta were found in yolk-sac larvae, and formed 5–7 days before hatching, but asteriscus were not found until 11 days post-hatching. Both the lapillus and sagitta were easy to handle and used for the analyses.

All otoliths were extracted with fine dissection needles under a stereo-dissecting microscope, cleaned in ETOH and air dried. Otolith increments from yolk-sac larvae were counted directly from the whole otolith, whereas those of post-yolk-sac larvae required polishing. Under the dissecting microscope, the sulcus side of the otolith was mounted downward with thermoplastic glue to a glass slide, and the mounted otolith was polished with 1500 grit wet-dry carbide sandpaper until the increments were countable. Increments were counted from the core to the outer edge with a light microscope at magnifications of 400× or 1000×. Each otolith was blind-counted three times by the same person at an interval of over 1 week. If two or three readings agreed and none of the counts differed by more than one, the sample was accepted for the counting the increments (Pfeiler et al., 2000). If the sample had three different readings or any two counts differentiated more than one, it was discarded.

### Statistical analysis

Ordinary least squares regressions were used to test the relationships of total length versus otolith radius (lapilli and sagittae), and increment counts of lapilli/sagittae versus the days post-hatching/the days post-marking. *Z*-tests were used to examine whether the regression lines of increment counts of lapilli/sagittae versus the days post-hatching/the days post-marking were different from regression line with slope 1 and intercept 0. All the data processing and analyses were performed in OriginPro 8.0 (OriginLab Corporation, Northampton, MA, USA).

## Results

The total length for newly hatched larvae was 9.23 ± 0.39 mm. In Experiment 1, the mean growth rate was 0.482 ± 0.107 mm day^−1^. The larvae lay on the bottom of the tubs on the first day after hatching, swam upward occasionally on the second day and swam intermittently in parallel on the seventh day. Yolk was exhausted about 11 days after hatching,.

**Figure 2:**
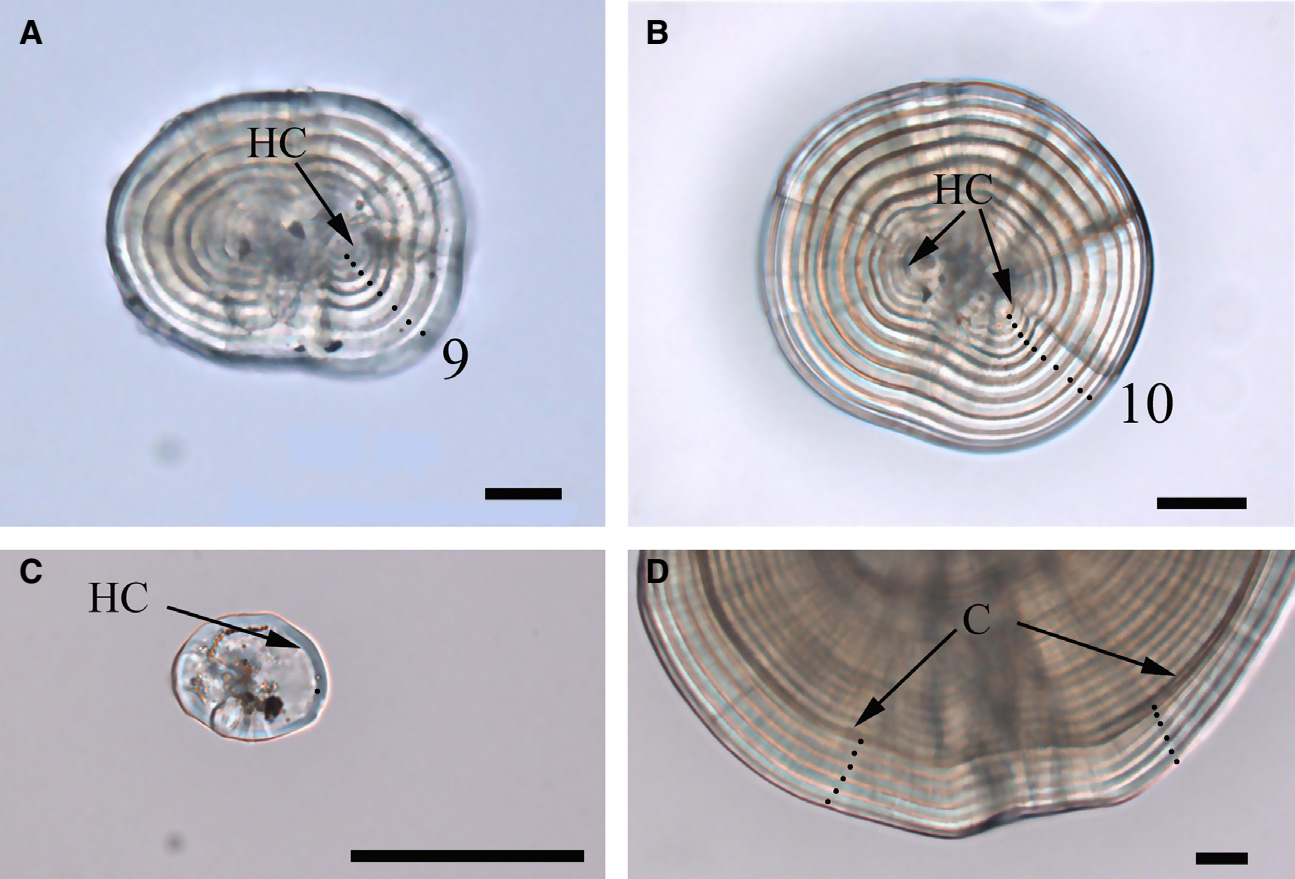
Otoliths of larval *Gymnocypris selincuoensis*. (A) A lapilli from a hatchery-reared yolk-sac larva showing the hatch check (HC) and subsequent daily increments, (B) A sagitta from a hatchery-reared yolk-sac larva showing the hatch check (HC) and subsequent daily increments, (C) A sagitta from a newly hatched larva showing the first increment as the hatch check (HC), (D) A sagitta from a wild-caught post-yolk-sac larva, showing a discernible check (C) and subsequent daily increments. Scale bar = 10 *μ*m

Otolith radius (lapillus, *R*_L_; sagtta, *R*_s_) increased linearly with total length (*T*_L_ = 0.094*R*_L_ + 8.78, *r*^2^ = 0.82, *n* = 70; *T*_L_ = 0.089*R*_S_ + 8.34, *r*^2^ = 0.86, *n* = 70); this demonstrated that somatic and otolith growth were isometric at this life stage. The increments on sagitta and lapillus of the yolk-sac larvae were distributed in concentric circles which were easily quantified (Fig. 2A–C), the width of increments on otoliths from reared larvae and wild larvae were showed on Figure1.

**Figure 3:**
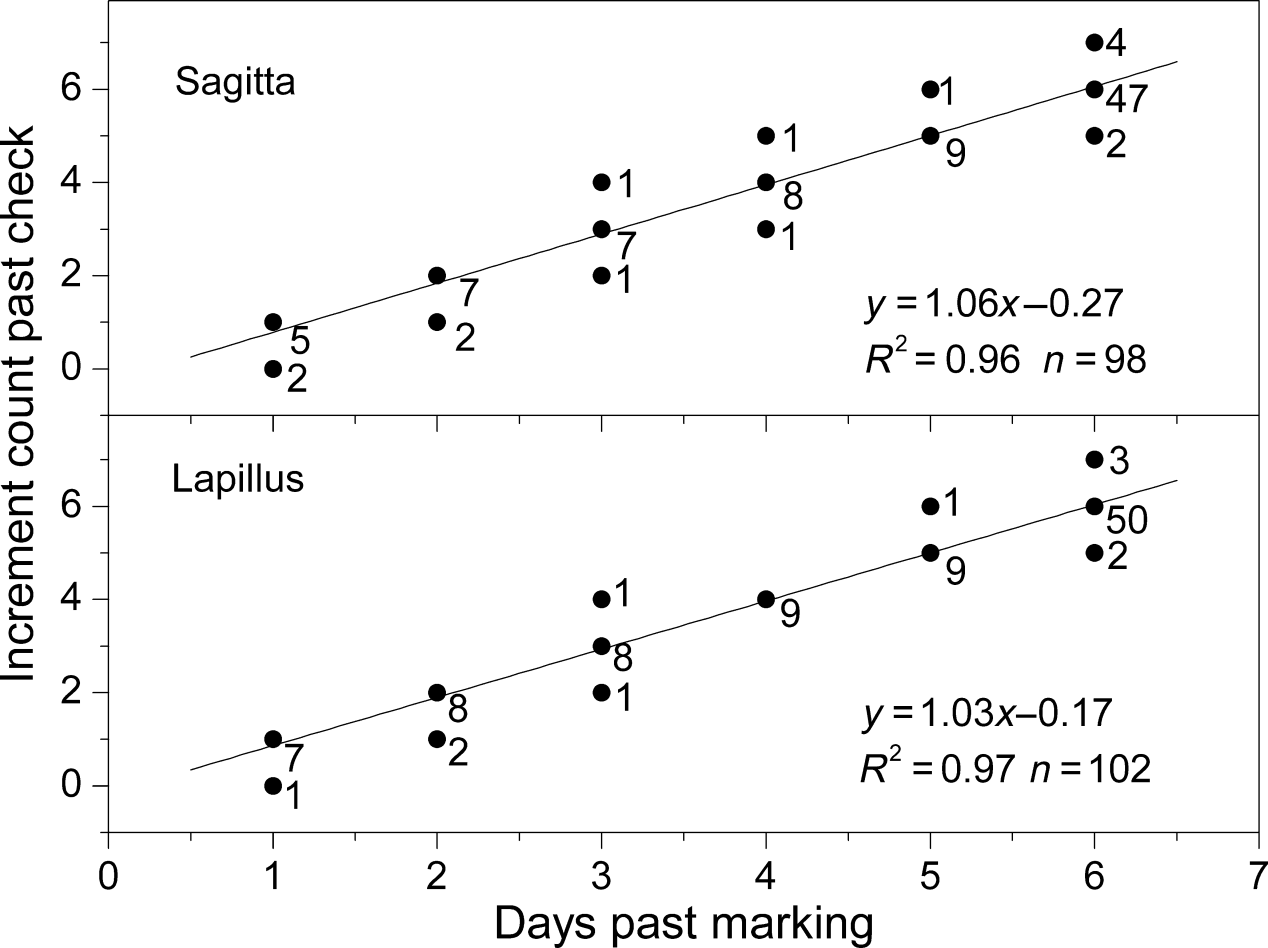
Linear regression between the otolith increment counts and the days post-hatching in yolk-sac larvae of *Gymnocypris selincuoensis*. The numbers to the right of the dots indicate numbers of specimens.

The regression between the otolith increment counts and the days post-hatching in yolk-sac larvae was expressed as a linear relationship using ordinary least squares regression analyses (Fig. 3). The curves were fitted by: *y* = 0.04 + 1.05*x* for lapillus (95%CI for intercept = −0.54 to 0.63 and slope = 0.96 to 1.14; *n* = 72, *r*^2^ = 0.85, *P* < 0.001) and *y* = 0.01 + 1.06*x* for sagitta (95%CI for intercept = −0.61 to 0.63 and slope = 0.97 to 1.15; *n* = 73, *r*^2^ = 0.82, *P* < 0.001). The slope for lapillus and sagitta were not significantly different from 1 (*Z*-test, *P* > 0.05), confirming that increments on sagitta and lapillus in yolk-sac larvae were deposited on a daily basis and had the intercept for lapillus and sagitta were not significantly different from 0 (*Z*-test, *P* > 0.05), confirming that the first increment on sagitta and lapillus were formed on the day of hatching. Although increment counts of 26.3% sagittae and 20.8% lapilli were older than the actual age of samples by 1–4 days, most of the increment counts were consistent with the actual age of samples.

**Figure 4:**
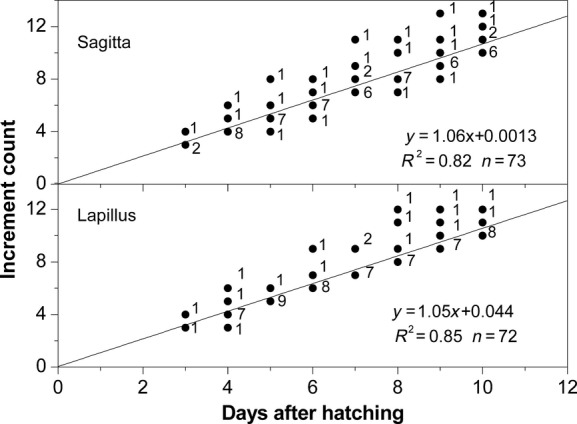
Linear regression between the otolith increment counts and the days post-marking in post-yolk-sac larvae of *Gymnocypris selincuoensis*. The numbers to the right of the dots indicate numbers of specimens.

Among 106 wild-caught post-yolk-sac larvae, 96.2% lapilli and 92.5% sagittae had a distinct narrow increment when viewed under a light microscope, which corresponded to the capture date (Fig. 2D). The regressions between the increment counts and the days post-marking was expressed as a linear relationship using ordinary least squares regression analyses (Fig. 4). The curves were fitted by: *y* = −0.17 +1.03*x* for lapillus (95%CI for slope = 0.99 to 1.07; *n* = 102, *r*^2^ = 0.97, *P* < 0.001) and *y* = −0.27 + 1.06*x* for sagitta (95%CI for slope = 0.99 to 1.12; *n* = 98, *r*^2^ = 0.96, *P* < 0.001). Again the slopes for lapillus and saggita were not significantly different from 1.0 (*Z*-test, *P* > 0.05), confirming daily periodicity of increments deposition in both the sagitta and lapillus from post-yolk-sac larvae.

## Discussion

The results showed that both sagittae and lapilli of yolk-sac larval and post-yolk-sac larval are regular in shape and well-suited for age determination. However, sagittae of individuals at earlier days (<50 days) have wider daily increments which make them easier and better for age determination than lapilli. For larvae older than 50 days, sagittae become elongateas in ostariophysans species (Hoff et al. 1997; Morioka and Matsumoto 2007), producing fragile rostrum structures which are inappropriate for increment analyses (unpublished data). Therefore, the lapillus is the only otolith pair suitable for daily age determination of the larval *G. selincuoensis* older than 50-day.

Examinations of otoliths from known-age or from marked individuals are probably the most rigorous and reliable methods for increments validation (Geffen 1992). In this study, both methods were adopted in validation of daily increment formation of otoliths from yolk-sac larae and post-yolk-sac larvae. In Experiment 1, the *G. selincuoensis* has been artificial propagated and hatched successfully. Therefore, we monitored known-age larvae to validate daily increment formation in otoliths of this species. The results showed that increments in lapillus and sagitta were daily deposited during the yolk-sac larvae stages of this species. The first increment was formed on the first day after hatching, which was consistent with some other species of Cypriniformes (Xie et al. 1995; Song et al. 2008).

Marking otoliths is probably the best method for validation in unknown-age fishes (Geffen 1992). Fish otoliths can be marked with chemical compounds and environmental stress. Many studies demonstrated the otoliths checks which marked with chemical compounds can be used for increments validation. However, there have been some debate about which chemical compound is the most efficient and least toxic, for some chemical compound and treatments may induce to high mortality rates (Geffen 1992). Alternatively, environmental stress such as capture stress, handling stress, temperature fluctuations and short photoperiod cycles can also mark otoliths and present a distinctive check on otoliths (Marshall and Parker 1982; Geffen 1983; Fowler 1989; Wright 1991). However, few studies have utilized these checks to validate the periodicity of increment formation, only Volk et al. (1984) and Boehlert and Yoklavich (1985). They used otoliths checks induced by handling and transfer stress as temporal markers for increment validation, though these approaches have not been tested systematically. In this study, environmental stress associated with capture and transportation, as expected, created a discernible check in 96.2% and 92.5% of lapilli and sagittae which proved to be a temporal marker for wild *G. selincuoensis* larvae. This finding was promising because it indicated that this marking method is feasible for future applications. Furthermore, compared with other routine marking methods, such as marking with chemical compounds, this method is simple in operation and no significant mortality has been detected during the process. In view of the advantages of this marking method, it is believed that this approach can be an alternative for otolith marking experiment for other fishes.

Under experiment conditions, the increments both in the lapillus and sagitta of yolk-sac larvae and post-yolk-sac larvae were daily deposited. However, it is believable that the wild larvae may experience a variety of environmental factors such as photo periodicity, water temperature fluctuation, hunger, and these factors can change the increment width and even deposition periodicity of increments. Consequently, it is very important to replicate such natural conditions for validation experiments (Geffen^1992^). In the present study, the daily rhythm of increments deposition was validated under controlled conditions very similar to the wild, except that the mean water temperature in laboratory was slightly higher than that in the field (Fig. 1). Under the experimental conditions, the daily growth increment width of laboratory-reared larvae is larger than those of wild larvae (Fig. 1), indicating that the water temperature has positive effect on the daily growth increment width. For wild larvae, the narrowest increment of the sagitta and lapillus formed at the lowest temperatures (5.5oC and 6.1oC, wild survey), were 1.28 and 1.07 *μ*m respectively (unpublished data). They are still wider than the resolution range of light microscopy and easily distinguishable (Campana et al. 1987). Therefore, it is reasonable to assume that the daily rhythm of increments deposition validated in this study is well suited for wild larval *G. selincuoensis* which experienced lower temperatures.

The validation of the first increment formation time and daily periodicity of increment deposition in the otoliths of *G. selincuoensis* is valuable in its early-life history and recruitment studies. Although the periodicity of deposition was validated under semi-natural conditions, the results should be applicable to wild fish (Geffen 1992). In addition, this study validated the daily increment formation in Schizothoracine fish for the first time has primary implications to other fishes from this subfamily.
